# Silica coated high performance oxide ceramics promote greater ossification than titanium implants: an in vivo study

**DOI:** 10.1186/s13018-022-03494-7

**Published:** 2023-01-11

**Authors:** Filippo Migliorini, Hanno Schenker, Marcel Betsch, Nicola Maffulli, Markus Tingart, Frank Hildebrand, Sophie Lecouturier, Björn Rath, Jörg Eschweiler

**Affiliations:** 1 Department of Orthopaedic, Trauma, and Reconstructive Surgery, RWTH University Hospital, Pauwelsstraße 30, 52074 Aachen, Germany; 2 Department of Orthopaedic and Trauma Surgery, Eifelklinik St. Brigida, 52152 Simmerath, Germany; 3 Department of Orthopedics, University Hospital of Erlangen, 91054 Erlangen, Germany; 4 Department of Medicine, Surgery and Dentistry, University of Salerno, 84081 Baronissi, SA Italy; 5 Faculty of Medicine, School of Pharmacy and Bioengineering, Keele University, Stoke-on-Trent, ST4 7QB UK; 6 Barts and the London School of Medicine and Dentistry, Queen Mary University of London, Mile End Hospital, 275 Bancroft Road, London, E1 4DG UK; 7 Ortho-Centrum Aachen (OCA), Aachen, Germany; 8 Department of Orthopaedic Surgery, Klinikum Wels-Grieskirchen, 4600 Wels, Austria

**Keywords:** High performance oxide ceramics, Implantology, Ossification

## Abstract

**Background:**

This in vitro study investigated the osseointegration and implant integration of high performance oxide ceramics (HPOC) compared to titanium implants in rabbits.

**Methods:**

Histomorphometry was conducted around the distal, proximal, medial, and lateral aspects of the HPOC to quantify the amount of mature and immature ossification within the bone interface. Histomorphometry was conducted by a trained musculoskeletal pathologist. The region of interest (ROI) represented the percentage of surrounding area of the implant. The percentage of ROI covered by osteoid implant contact (OIC) and mature bone implant contact (BIC) were assessed. The surrounding presence of bone resorption, necrosis, and/or inflammation were quantitatively investigated.

**Results:**

All 34 rabbits survived the 6- and 12-week experimental period. All HPOC implants remained in situ. The mean weight difference from baseline was + 647.7 mg (*P* < 0.0001). The overall OIC of the ceramic group was greater at 6 weeks compared to the titanium implants (*P* = 0.003). The other endpoints of interest were similar between the two implants at all follow-up points. No difference was found in BIC at 6- and 12-weeks follow-up. No bone necrosis, resorption, or inflammation were observed.

**Conclusion:**

HPOC implants demonstrated a greater osteoid implant contact at 6 weeks compared to the titanium implants, with no difference found at 12 weeks. The percentage of bone implant contact of HPOC implants was similar to that promoted by titanium implants.

## Introduction

Alloy implants are commonly used in musculoskeletal medicine [1]. Bony osteointegration is pivotal to ensure implant survivorship. Osseointegration is a foreign body reaction to shield off the implant from the tissues [2]. Osteointegration is the capability of the implant to fuse with the surrounding bone, interacting and integrating with it [3, 4]**.** The rate of aseptic loosening and related consequences (stress-shielding, persistent pain, inflammation) are still a concern in musculoskeletal medicine following implantation of foreign materials [5, 6]. Given their optimal ossification, titanium and its alloys are commonly used [7–9]. High performance oxide ceramics (HPOC) has attracted much interest [10, 11]. HPOC have several advantages: high hardness and wear resistance, light weight, outstanding resistance to creep and compressive stress, and avoid imaging artefacts [12–14]. However, the biologically inactivity of ceramics, and consequently limited potential for integration, may limit their application. To overcome this limitation to clinical application, HPOC biologically functionalised with a silicate coating alumina AL_2_O_3_ have been developed [15, 16]. HPOC implants have been recently introduced in musculoskeletal medicine, and their application has been successfully supported by recently published clinical trials [17–24]. HPOC have been developed and investigated at our institution during the past years. The strength of the bonding between the functionalised ceramic and surrounding layer was evaluated by the application of polymethyl methacrylate (PMMA) to the surface, as per manufacturer instructions [25]. The contact guidance, adhesions, surrounding mesenchymal stromal cells osteogenic differentiation, and their cytotoxic potential has also been evaluated in previous studies conducted at our institution [25, 26]. An experimental study in rabbits was conducted to characterise osseointegration and implant integration of HPOC. The bone implant interface is responsible for the primary and secondary stability of the implant, and it is suggested to be the weakest domain in the bone implant system, where most failures occur [27, 28]. We hypothesised that HPOC implants promote ossification and transplant integration. To validate this hypothesis, osteointegration of HPOC were compared to that provided by commercial titanium implants. Histomorphometry was conducted around the distal, proximal, medial, and lateral aspect of the HPOC implants to quantify the amount of mature and immature ossification within the bone implant interface.

## Materials and method

### Type of implants

For the experimental group, HPOC were manufactured and functionalized at the RWTH University Aachen, Germany, Department of Dental Materials Science and Biomaterial Research in a previously published fashion [25, 26, 29, 30]. Briefly, standard 5.5 × 8 mm Al_2_O_3_ ceramic based cylinders were used. To facilitate the coupling of stable organosilane monolayers on the monolithic Al_2_O_3_ ceramic based cylinders, plasma-enhanced chemical vapor deposition (PE-CVD) was performed [31]. Silicon suboxide (SiOx) was deposited on the polished and cleaned Al_2_O_3_ ceramic based cylinders to activate the ceramics [32]. Functionalised ceramic cylinders were then air-dried, cured at 80° for 45 min, and stored in liquid nitrogen until use. For the control group, Sandblasted titanium Ti-6Al-4 V 5.5 × 8 mm implants (Zimmer Biomet GmbH, Neu-Ulm, Germany) were used.

### Surgical procedure

**Fig. 1:**
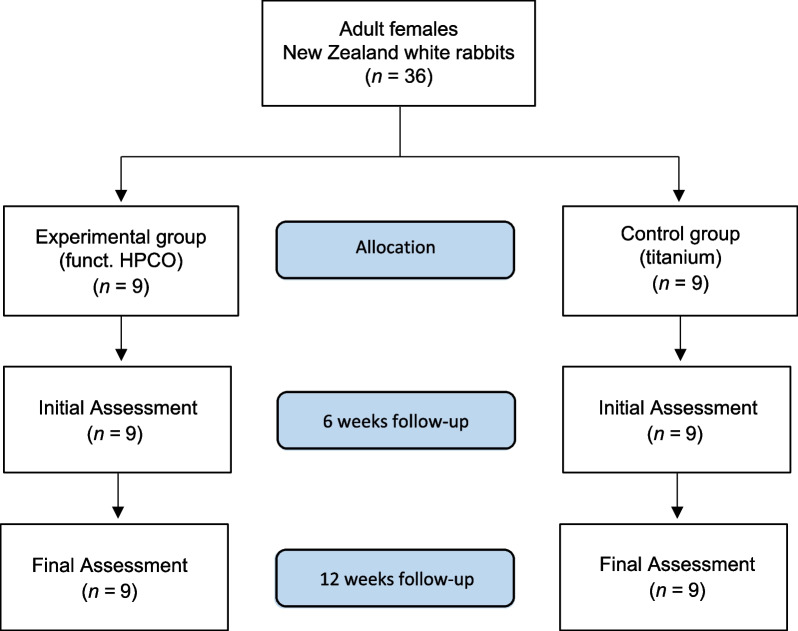
Group allocation

The present investigation was conducted following the Animal Welfare Act of the Federal Republic of Germany in 2017, and approved by the Federal Office for Nature, Environment and Consumer Protection (Landesamt für Natur, Umwelt und Verbraucherschutz, LANU) of North Rhine-Westphalia, Federal Republic of Germany (Approval ID: 84–02.04.2016.A434). 34 New Zealand adult female white rabbits (weight > 3 kg) were used. Rabbits were allocated into four groups (Fig. 1).

0.1 ml/mg/kg bodyweight Medetomidin (Domitor) combined with a 0.2 ml Ketamin (Narketan) in a subcutaneous injection were used to induct the anaesthesia. The surgical site was shaved, disinfected with iodine and ethanol, and draped in a sterile fashion. 10 mg/kg bodyweight Enrofloxacin subcutaneous injection were injected before the incision. The skin incision was performed over the right lateral femoral condyle. After dissection through fascia and muscles, the condyle was exposed, and the lateral collateral ligament identified. With a 5.5 mm trephine, a unicortical drill hole was performed under irrigation to avoid thermal necrosis, making sure that the lateral collateral ligament was sparred. Once the bony cylinder was extracted, either a titanium or HPCO cylinder was inserted in a press fit fashion, without damaging the knee capsule. Tissues were closed in layers in a standard fashion. Finally, the skin was stapled and sprayed with chelated silver. For the first three days after surgery, 4 mg/kg bodyweight Carprofen were administered every 24 h. Six or 12 weeks postoperatively, the rabbits were euthanised using 2 ml/kg bodyweight Natriumpentobarbital (160 mg Natriumpentobarbital/ml). The femoral condyles were harvested and examined.

### Sample preparation

The femoral condyles were harvested en bloc. Fixation was performed over 12 days with 4% paraformaldehyde followed by an alcohol series with ethanol of 50–100% and xylol. The specimens were embedded in Technovit^®^ 9100 (Kulzer GmbH, Hanau, Germany). Using a diamond band saw Exakt 300CL (EXAKT Technologies Inc, Oklahoma City, US), 60–70 µm coplanar cuts of the specimens were performed. Grinding of titanium cylinders was conducted using sandpaper, while HPCO implants were ground with diamond paper. All specimens were stained with haematoxylin eosin, trichrome, and toluidine. Histomorphometry was conducted by a professional pathologist with OLYMPUS digital microscope DSX-1000 and stream desktop- Software (Olympus Hamburg, Germany).

### Histomorphometry

**Fig. 2:**
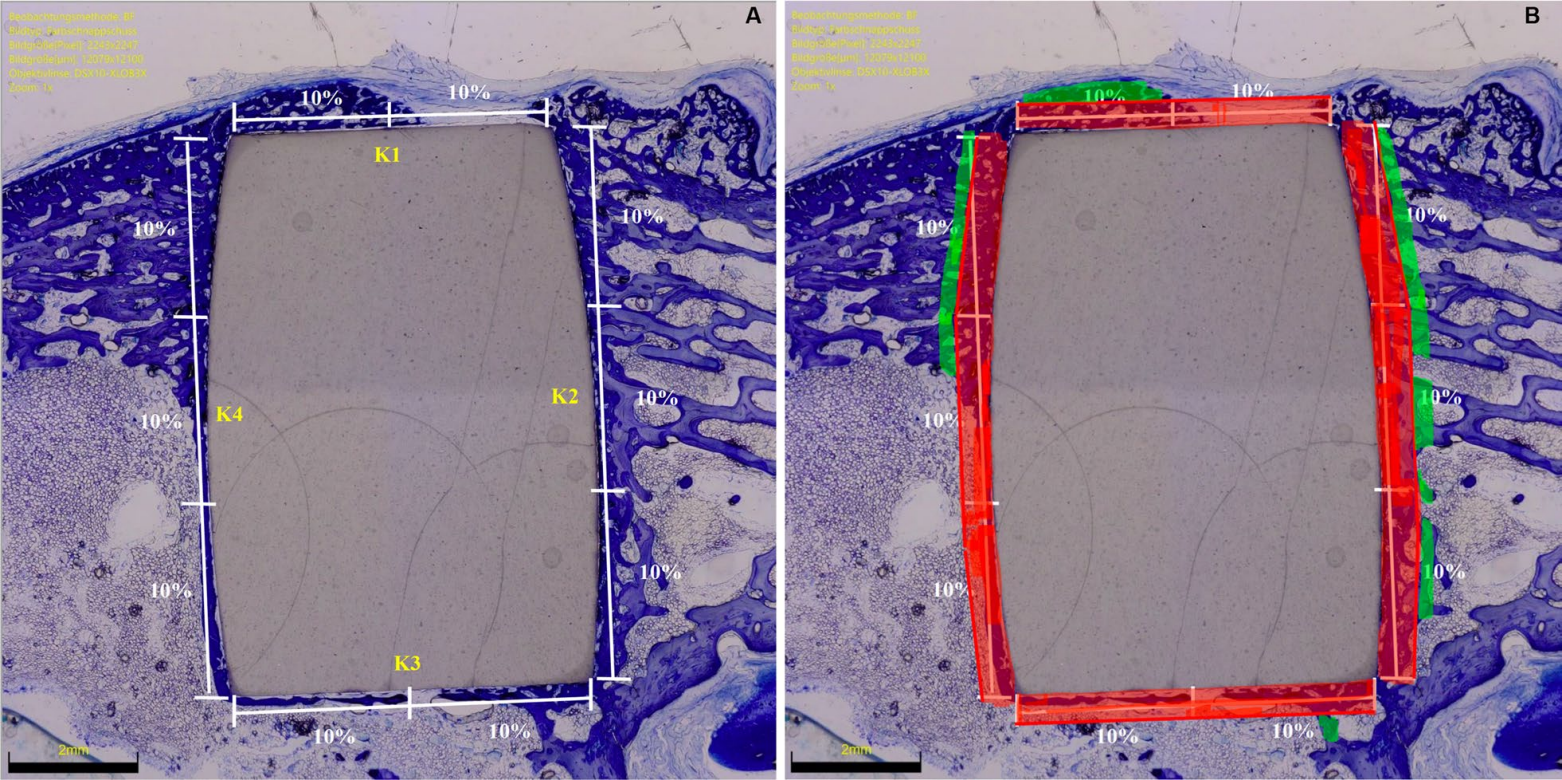
Left: Microscopy evaluation strategy of the BIC: K2 and K4 (longer sides) accounted for 60% (30% each) and the K1 and K3 (shorter sides) for 40% (20% each). Right: Region of interest

At microscopy, specimens were divided as follows: lateral (K1), distal (K2), medial (K3), and proximal (K4). The surrounding area between the bone-implant interface (red zones) was the region of interest (ROI, Fig. 2).

The outcome of interest was to investigate the potential of osteointegration of HPCO in comparison to the standard titanium. Hence, the percentage immature, and unmineralized bone matrix (osteoid implant contact, OIC) within the ROI was assessed. The percentage of mature, mineralized bone (bone implant contact, BIC) within the ROI was also quantified. The surrounding presence of bone resorption, necrosis, and/or inflammation were quantitatively evaluated and classified as follows: 0 (none), 1 (minimal), 2 (low), 3 (moderate), 4 (severe).

### Statistical analysis

The IBM SPSS (version 25) was used for statistical analyses. Mean and standard deviation were used for descriptive statistics. The mean difference effect measure was adopted for continuous variables, with standard error (SE) and 95% confidence interval (CI). Values of *t*-test < 0.05 were considered statistically significant.

## Results

### Animal data

All 34 rabbits survived the 6- or 12-week experimental period. Four wounds dehiscence were further stapled without any signs of wound infection. No rabbit died during the experimental period. At euthanasia, no clinical signs of inflammation or adverse tissue reactions were observed. All implants remained in situ. At baseline, rabbits had a mean weight of 3254.2 ± 199.6 mg. At last follow-up, rabbits had a mean weight of 3901.9 ± 275.0 mg. The mean weight difference from baseline was + 647.7 mg (*P* < 0.0001).

### Result syntheses

**Fig. 3:**
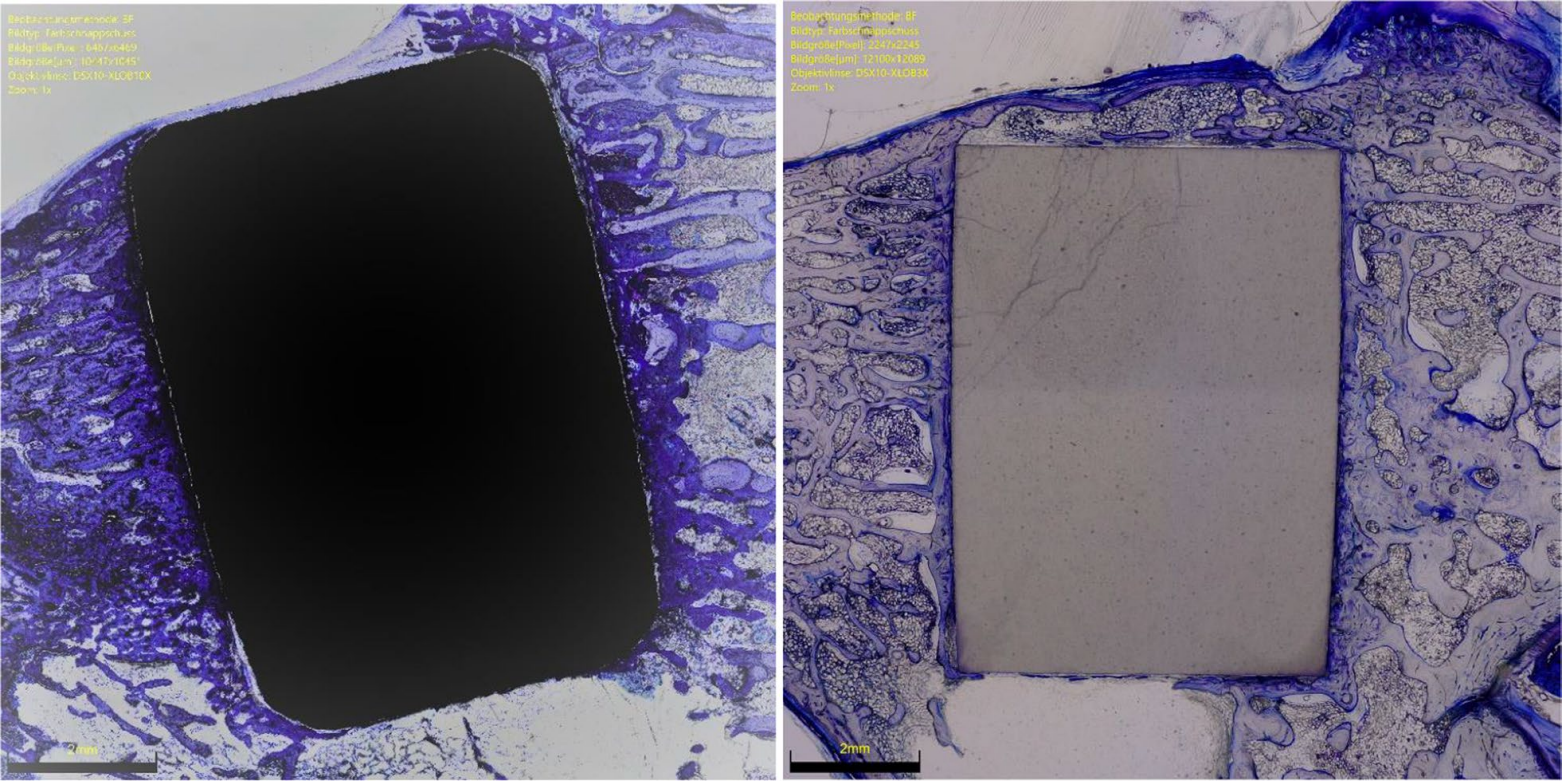
Titanium (*left*) and ceramic (*right*) implants at 6 weeks (magnification: 1x). Toluidine blue staining

**Fig. 4:**
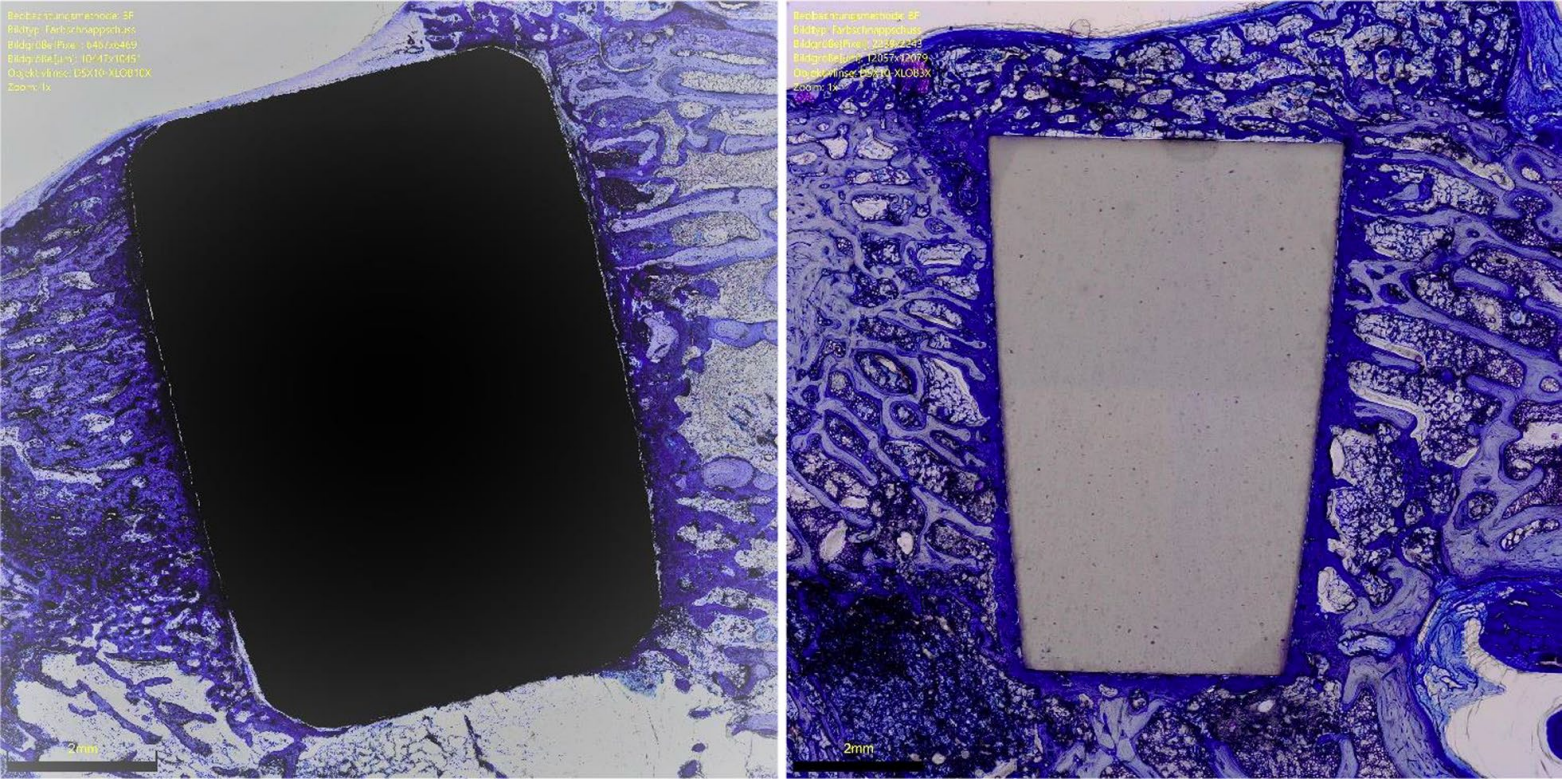
Titanium (*left*) and ceramic (*right*) implants at 12 weeks (magnification: 1x). Toluidine blue staining

**Fig. 5:**
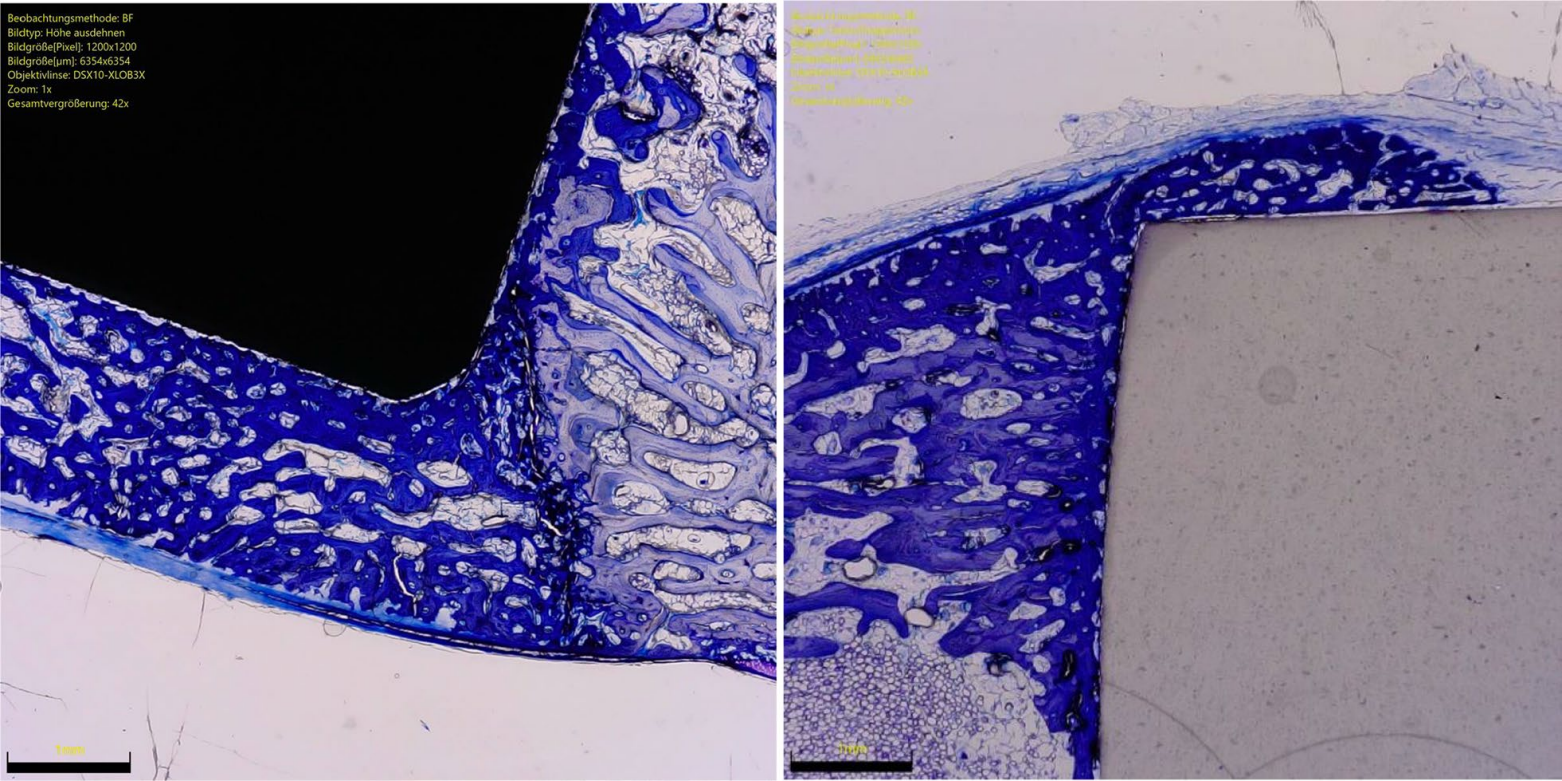
Titanium (*left*) and ceramic (*right*) implants at 12 weeks (magnification: 42x). Toluidine blue staining

**Fig. 6:**
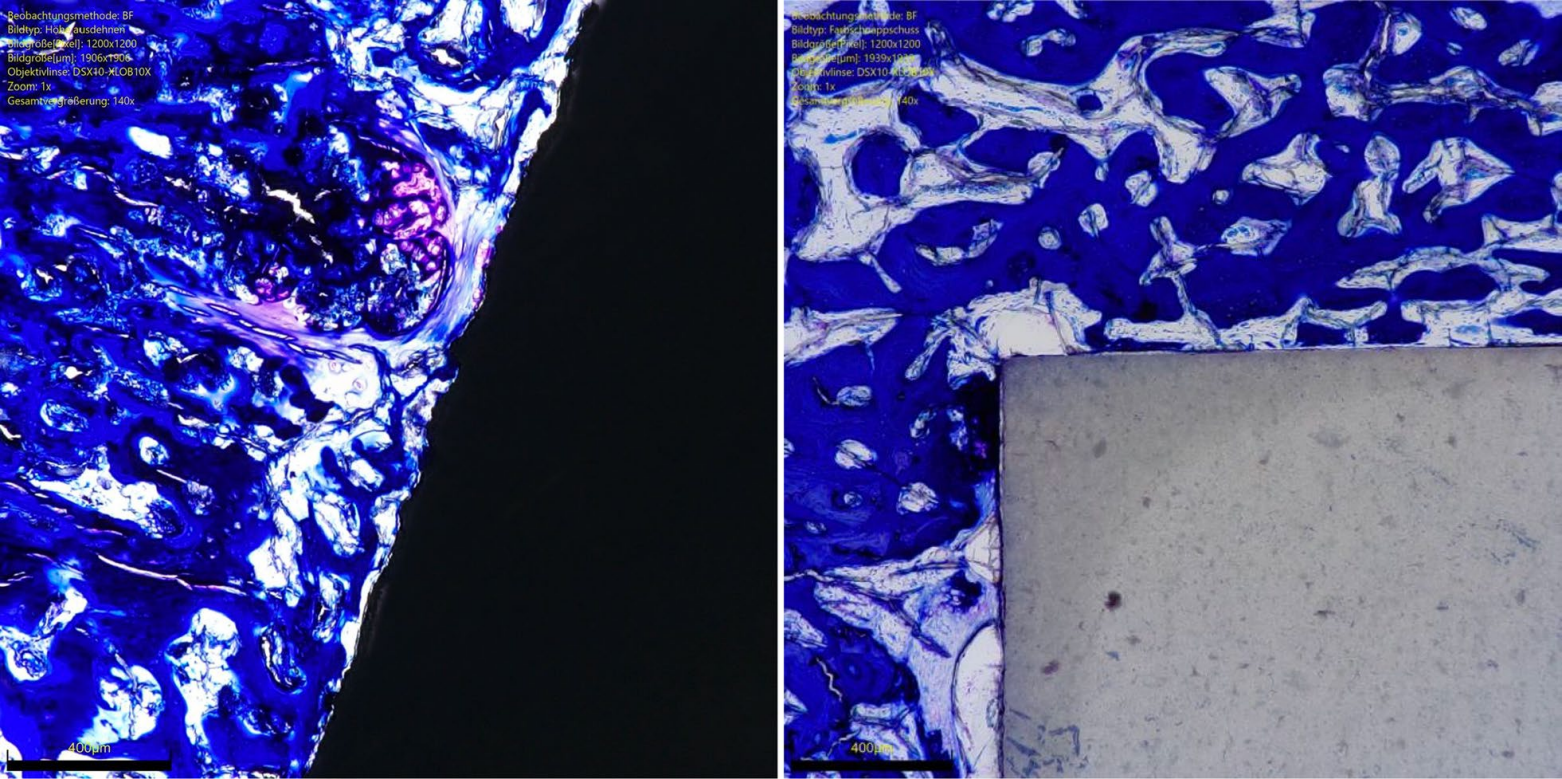
Titanium (*left*) and ceramic (*right*) implants at 12 weeks (magnification: 140x). Toluidine blue staining

**Fig. 7:**
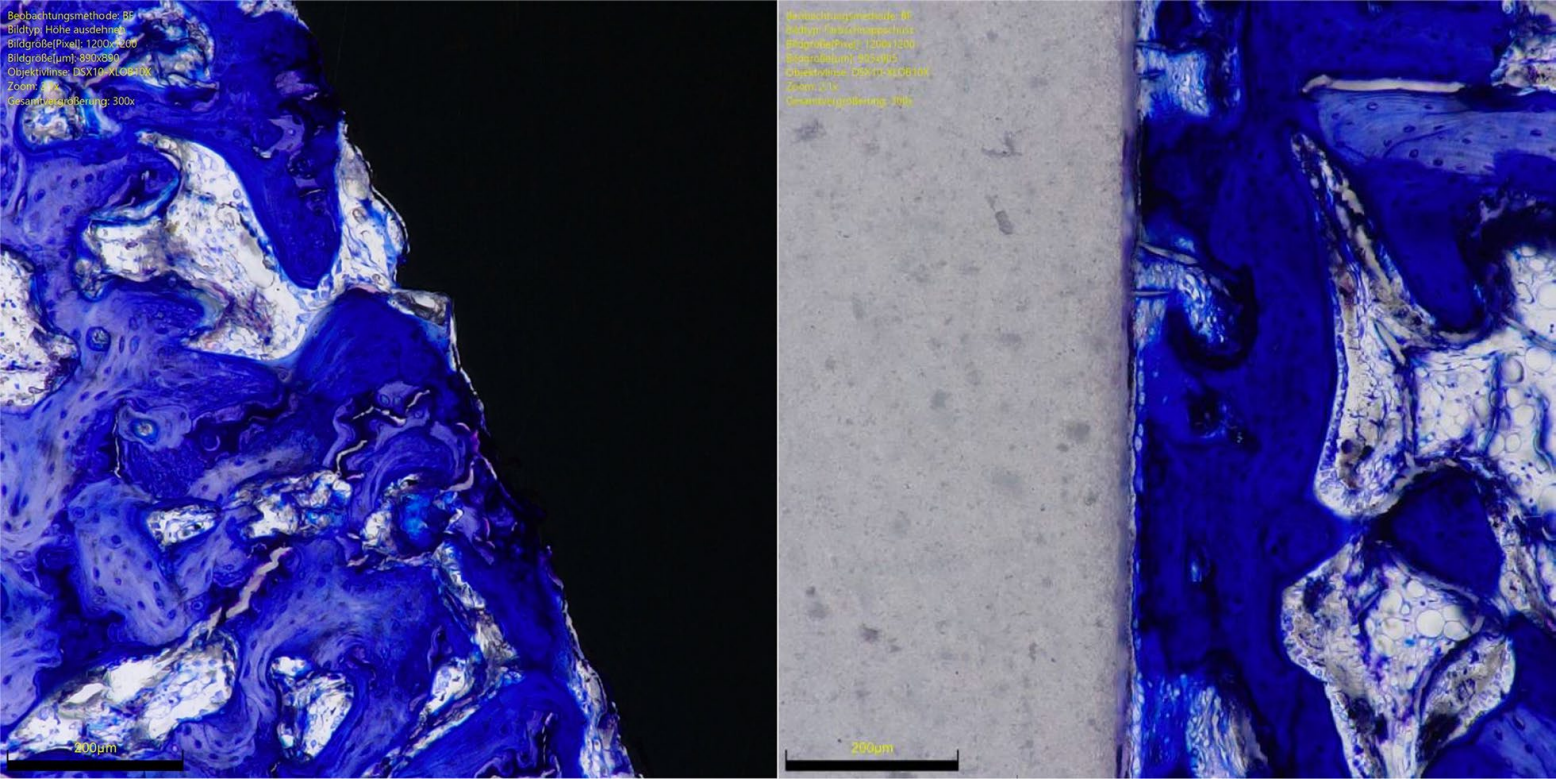
Titanium (*left*) and ceramic (*right*) implants at 12 weeks (magnification: 300x). Toluidine blue staining

**Fig. 8:**
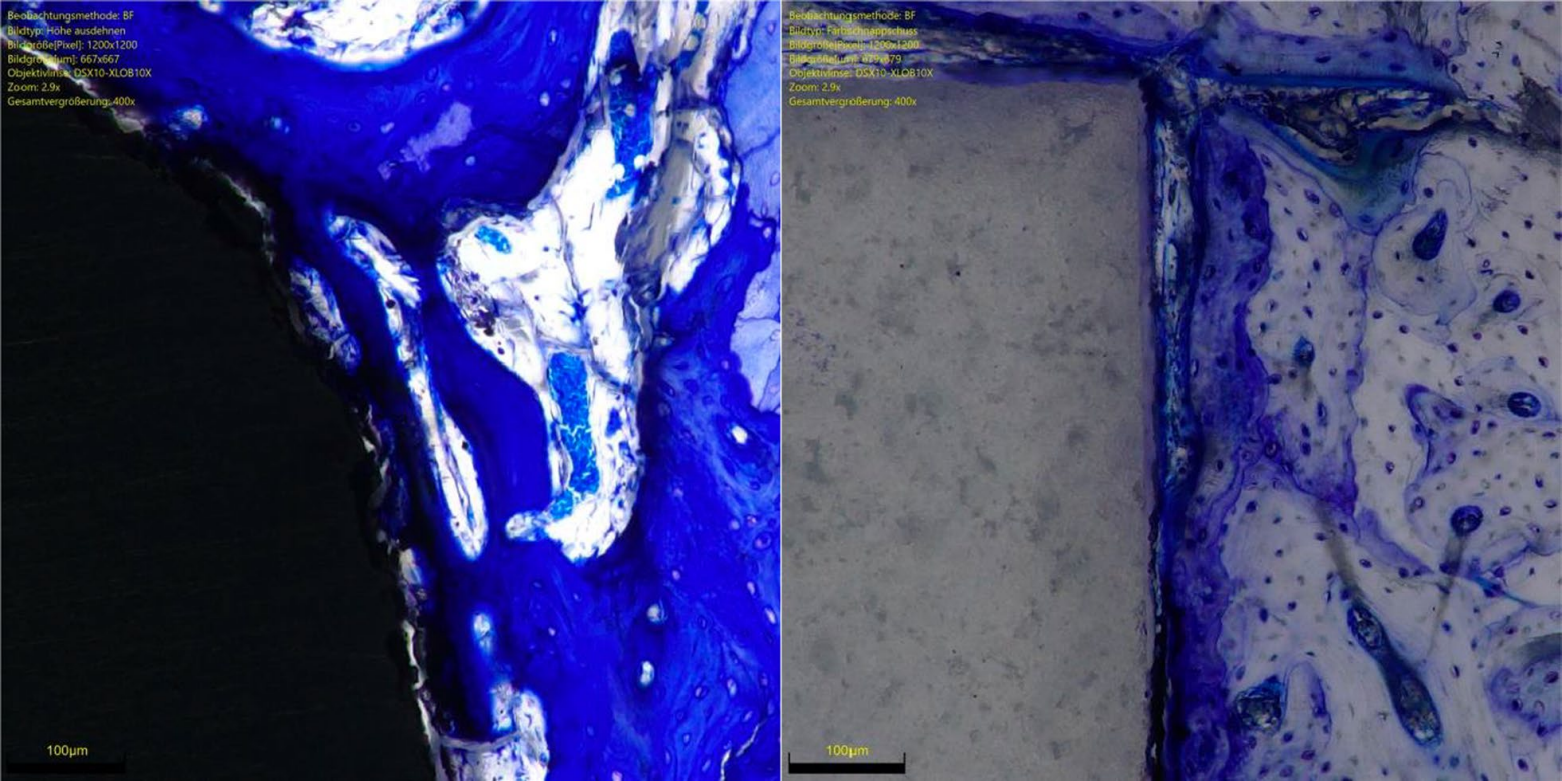
Titanium (*left*) and ceramic (*right*) implants at 12 weeks (magnification: 400x). Toluidine blue staining

The overall OIC of the ceramic group was greater at 6 weeks compared to the titanium implants (*P* = 0.0001). The other endpoints of interest were similar between the two implants at both follow-up times (*P* ≥ 0.05). No difference was found in BIC at 6- and 12-weeks. The results of the quantitative analyses are shown in detail in Table 1. Figures 3 and 4 show an overview at 1 × magnification of the implants at six and 12 weeks, respectively. Figures 5, 6, 7 and 8 show respectively an overview at 42 × , 140 × , 300 × , 400 × magnification of the implants at 12 weeks.

**Table 1:** Comparison of HPOC versus titanium at 6- and 12-weeks follow-up (MD: mean difference)

Endpoint		6 weeks				12 weeks			
		Ceramic	Titanium	MD	*P*	Ceramic	Titanium	MD	*P*
*Lateral*									
BIC (%)		3.0 ± 2.6	3.1 ± 3.9	− 0.1	0.5	2.8 ± 3.5	3.9 ± 3.7	− 1.0	0.3
OIC (%)		0.9 ± 1.7	0.0 ± 0.3	0.9	0.07	1.6 ± 2.1	0.4 ± 0.1	1.1	0.09
*Distal*									
BIC (%)		13.2 ± 6.6	11.5 ± 2.1	1.7	0.2	13.9 ± 7.6	13.6 ± 7.7	0.2	0.5
OIC (%)		5.8 ± 2.5	1.6 ± 3.5	4.2	*0.003*	5.6 ± 4.6	5.0 ± 3.6	0.6	0.4
*Medial*									
BIC (%)		5.6 ± 3.7	5.3 ± 3.1	0.2	0.4	8.2 ± 4.5	7.1 ± 3.5	1.1	0.3
OIC (%)		2.2 ± 1.5	− 0.3 ± 1.9	2.5	*0.003*	2.8 ± 2.3	1.4 ± 1.4	1.3	0.1
*Proximal*									
BIC (%)		12.0 ± 6.2	10.8 ± 8.7	1.2	0.4	12.6 ± 6.2	11.6 ± 3.9	1.0	0.4
OIC (%)		5.0 ± 0.0	5.0 ± 0.9	0.0	1.0	5.0 ± 0.0	4.3 ± 2.4	0.7	0.1
*Overall*									
BIC (%)		0.3 ± 0.1	0.3 ± 0.1	0.0	0.4	0.4 ± 0.1	0.3 ± 0.7	0.0	0.2
OIC (%)		13.9 ± 3.8	6.3 ± 1.7	7.6	*0.0001*	14.9 ± 7.7	11.1 ± 6.1	3.8	0.2
BIC (%)		0.6 ± 0.2	0.6 ± 0.6	0.0	0.5	0.3 ± 0.1	0.4 ± 0.4	0.0	1.0

There is evidence of greater osteoid ossification in the ceramics group compared to the titanium cohort only at 6 weeks in the distal (*P* = 0.003), medial (*P* = 0.003) sides and at the overall level analysis (*P* = 0.0001)

No other difference between the two implants has been evidenced in the other endpoints at both 6 or 12 weeks

## Discussion

The present study investigated the integration potential of HPOC implants, and then compared in vivo to titanium implants at 6 and 12 weeks. HPOC implants promote similar osteointegration to commercial titanium implants in rabbits. HPOC implants demonstrated greater osteoid implant contact at 6 weeks compared to the titanium implants, with no difference at 12 weeks. The percentage of bone implant contact of HPOC implants was similar to the titanium implants. No bone necrosis, resorption, nor inflammation was observed.

Bone is a dynamic tissue which undergoes continuous modifications and adaptations. For end stage degenerative or traumatic bony ailments, permanent or temporary implants are commonly used to restore bony function and quality of life. In the case of permanent implants (e.g. arthroplasty) the integration of the implant to the surrounding bone is crucial to ensure its longevity.

Recently, implants coating has attracted much interest. Titanium alloys may be responsible of hypersensitivity reactions, which may compromise implant longevity [33–38]. When low-grade infection and other mechanical problems have been excluded, symptoms such as pruritus, pain, effusion, erythema, hypersensitivity reactions should be taken into consideration [39]. Ions released by corrosion of metallic wear debris may impair ossification and metal particles can be found in the soft tissues surrounding the implant [40]. Particles and ions may become clinically relevant for sensitive patients. According to the 2016 Australian Arthroplasty Register, approximately 2% of revision TKAs are attributed to metal-related pathology [41]. In selected patients with hypersensitivity, non-metallic implant solution as possible savage revision is limited with unpredictable results [42].

Current researches to develop alternative to the metal alloys are ongoing. In this context, ceramics are attracting growing interest and broad researches. Implant coating aims to optimize the biological properties of the surface to improve ossification. Ceramics are biologically inert, and consequently do not promote implant ossification. However, ceramics have high hardness and wear resistance, light weight, outstanding resistance to creep and compressive stress, avoiding imaging artefacts [12–14]. The biological inactivity of ceramics also prevents to systemic reactions to ceramic wear debris and aseptic osteolysis. Therefore, following the development of novel coating methodologies, ceramic implants have triggered renewed interest [15, 16, 43, 44].

Several coating process to functionalise the surface of ceramics have been developed with promising pre-clinical and clinical results [45–50]. Previous studies conducted in rabbit models used heterogeneous coating methodologies, follow-up, analyses, and implantation sites (Table 2). The current evidence on the clinical applications of ceramics is still limited, and translational studies are required to clarify the best coating process.

**Table 2:** Previous studies conducted on rabbit models investigating ceramic coating methodologies

Author, Ref	Rabbits model	Rabbits (n) and implant site	Materials	Follow-up	X-Rays and MicroCT	Biomechanical evaluation	Histology, histomorphometry, immunohistochemistry, PCR
Gong et al. [51]	30 New Zealand rabbits	Right medial tibia epiphysis of the (*d* = 6 mm, ø = 3 mm)	Calcium silicate powder, RA-CPSC, MCP, Ca(H2PO4)2, and risedronate added into calcium silicate powder and homogeneously mixed respectively	8, 10 weeks	↑ Trabecular bone formation with X-ray in Group II in comparison to Group I after 10 weeks	–	↑ up-regulation of genes in Group II in comparison with Group I
Lozano et al. [52]	Osteoporosis induced rabbits	Medial and lateral distal femoral epiphysis	SBA15 and SiO_2._ The surface was chemically modified with an organic modification of silica walls with alkoxysilane, *n*-octyltriethoxysilane and functionalized by soaking the mesoporous in a solution of PTHrP in PBS	2 weeks	–	–	No inflammation. Increase in staining for PCNA, Runx2 antibody, osteopontin, and/or VEGF in rabbits in both Group II and IV
Luo et al. [53]	60 New Zealand rabbits	Sub-periosteal mandibular (4 mm × 5 mm × 10 mm)	Porous commercial HA was physically functionalized in surface with or without APN or Matrigel or combination of both	4 weeks	Increase in NBV in Group I than the other groups. Reduction in TRAP activity in Group I than other groups	Increase in compressive strength and elastic modulus in Group I than other groups	Reduction in Tb.Sp in Group I than other groups. Increase in BV/TV, Conn.D, Tb.Th and Tb.N. in Group I than other groups
Migliorini et al. [49]	36 female New Zealand white rabbits	Lateral femoral condyles	BMP-2 coated HPOC (group I) versus sandblasted titanium implants (group II)	6, 12 weeks			The bone implant contact of BMP-2 coated HPOC increased similarly to standard titanium implants
Migliorini et al. [50]	36 female New Zealand white rabbits	Lateral femoral condyles	RGD coated HPOC (group I) versus sandblasted titanium implants (group II)	6, 12 weeks			The bone implant contact of RGD coated HPOC increased similarly to standard titanium implants
Plaza et al. [54]	42 New Zealand white rabbits	Medial femoral condyles	Physical incorporation of fibronectin in HA bulk material by adding HA to a fibronectin solution of in PBS	1,2,5 days	Increase in Tb.Th in Group I in the area next the screw in comparison with area far from the device	–	Increase in cellularity at 24 h in Group I than the other Groups
Shen et al. [55]	43 New Zealand white rabbits	Femoral epiphysis	TNT were immersed in supersaturated Ca(OH)_2_ solution, Ca(NO_3_)_2_·4H_2_O (0.2 M) and (NH4)2HPO4 (0.2 M) solutions to create a coating of HA. TNT-HA was subsequently functionalized with Aln by immersion in Aln solution (physical absorption)	12 weeks	no dislocation and inflammation occurred. Increase in BV/TV and Tb.Th in Group IV and III than Group I and II. Increase in	Increase in interfacial strength in Group IV than other groups and in Group III than Group I and II	Increase in osteoid tissue in Group IV and III than other Groups
Wu et al. [56]	16 New Zealand White rabbits	Distal femur	Strontium enriched CPC in the solid phase and PCL	24 weeks	Increase in BV/TV, Tb.Th, Tb.N, connectivity density in Group III than Group I. Reduction of Tb.Sp, SMI and total porosity in Group III than Group I and Group II		
Yu et al. [57]	40 New Zealand white rabbits	Two implants (2-mm diameter, 10-mm depth) into each femur	Ti–6Al–4 V implants (ø10 × 2 mm) coated by means of plasma-spray technique with HA or CaSiO_3_ or zinc-modified calcium silicate (Ca_2_ZnSi_2_O_7_) at two different Zn contents	4, 8, 12 weeks	All parameters ↑ from 4 to 8 weeks. ↑ highest values of all parameters in Group V than all other Groups		No new bone in Group I. Small bone in Group II and III stating from 4 weeks. Strong new bone formation in Group IV and V and improved osteointegration
Gunnella et al. [58]	30 California rabbits	Not specified (5 mm wide and 4 mm)	A composite material of HA/TCP granules with or without Sr substitution	12 weeks	–	–	Greater mean trabecular area in Group I in comparison to other groups. Increase of NFkB, OPG, OC, BMP 2/4, COL-1α and IL-1 in Group III in comparison to Group IV

Risedronate Calcium Phosphate Silicate Cements (RA-CPSC), Mesoporous silica (SBA15), monocalcium phosphate (MCP), polymerase chain reaction (PCR), Parathyroid hormone-related protein (PTHrP), Adiponectin (APN), Hydroxyapatite (HA), phosphate-buffered saline (PBS), TiO_2_ nanotube (TNT), hydroxyapatite-TiO_2_ nanotube (TNT-HA), calcium phosphate ceramic (CPC), poly(ε-caprolactone) (PCL), tricalcium phosphate 70% (TCP), peptide bone morphogenic protein (BMP), Arg-Gly-Asp (RGD)

The current evidence on the clinical application of HPOC is limited. Alumina ceramic for talar replacement has been developed over the past 20 years as an alternative to ankle arthrodesis [59]. In 2015, Tanighuki et al. [17] published their results on 55 total talar replacements using customised HPOC, with improvement in range of motion and patient reported outcome measures [17]. Moreover, all patients returned to their activities of daily living with no complication [17]. Several studies investigated HPOC implants for total knee arthroplasty. Most studies used an alumina [18–21] or a mixed alumina/zirconia [22–24] femoral component and metal tibial component.

Few studies investigated the clinical outcomes of a fully alumina [60] or mixed alumina/zirconia [24] total knee implant. Clinical trials indicated that HPOC implants for total knee arthroplasty performed as well as conventional metallic components in improving patient reported outcome measures (PROMs) or inducing complications. In a recent systematic review investigating the efficacy and safety of HPOC implants for total knee arthroplasty in 14 clinical studies, wear and breakage of the prosthesis only occurred in three knees after following up for 18.1 years and in 3 patients after a minimum follow-up of 5 years, respectively [61]. The overall estimate of the rate of revision was 0.03% [61]. Despite the limited use of ceramic knee implants worldwide, and considering the advanced tribological features of the ceramic components, these components demonstrated a promising role in knee implant surgery.

This study has several limitations. The assessors (pathologist and surgeon) were not blinded to the nature of the implant, potentially increasing the risk of detection and performance biases and overestimating the results. A formal mechanical test to prove implant stability (e.g. pull-out torque test) was not conducted. However, previous reports demonstrated a definite association between pull-out torque test and histomorphometry [62–65]**.** The absence of physiological load on the prostheses also impairs the validity of the present conclusion, and clinical studies should be performed. Rabbits, being reproducible, low cost, and easy to handle, are commonly used as animal model. However, between species differences exist, and must be considered in clinical translation. Current evidence could benefit of longer follow-up investigation. The results of the present study should encourage further translational studies to verify the ossification potential of ceramics in a clinical setting. Biomaterial research underlined that is possible to design and develop new bone implants with geometry, and chemical characteristics able to improve bone in growth and prevent infection. Two time points (6 and 12 weeks) were used to evaluate osteointegration. In the current literature, heterogeneous time points for analysis of implant osteointegration are reported. Though bone can be already observed at one week after implantation, proper bone remodelling starts between 6 and 12 weeks with primary and secondary osteons forming compact bone [66, 67]. Though implant roughness is a key parameter for the in vivo performance of a bone integrating insert, this parameter was not analysed in the present investigation, and further studies are required to overcome this limitation. Future studies should improve antibiofilm and antibacterial strategies to the novel materials with complementarity between knowledge and expertise in biology, chemistry, engineering and physics, associating biological models with material sciences and other technologies.

## Conclusions

HPOC implants demonstrated a greater osteoid implant contact at 6 weeks compared to the titanium implants, and no difference at 12 weeks. The percentage of osteoid production and bone implant contact of HPOC implants was similar to that observed in titanium implants. No bone necrosis, resorption, or inflammation were observed.

## Data Availability

The data presented in this study are available on request from the corresponding author.

## Abbreviations

RA-CPSC: Risedronate calcium phosphate silicate cements
SBA15: Mesoporous silica
MCP: Monocalcium phosphate
PCR: Polymerase chain reaction
PTHrP: Parathyroid hormone-related protein
APN: Adiponectin
HA: Hydroxyapatite
PBS: Phosphate-buffered saline
TNT: TiO_2_ nanotube
TNT-HA: Hydroxyapatite-TiO_2_ nanotube
CPC: Calcium phosphate ceramic
PCL: Poly(ε-caprolactone)
TCP: Tricalcium phosphate 70%
SE: Standard error
CI: 95% Confidence interval
PE-CVD: Plasma-enhanced chemical vapor deposition
SiO_x_: Silicon suboxide
HPOC: High performance oxide ceramics
PMMA: Polymethyl methacrylate
